# Invasive Fusariosis: Unusual Cases over 10 Years in a Tertiary Care Hospital and a Review of the Literature from Saudi Arabia

**DOI:** 10.3390/idr18010014

**Published:** 2026-01-26

**Authors:** Hassan Almarhabi, Abdulmajeed Sarhan, Murad Essatari, Hassan Huwait

**Affiliations:** 1 Department of Medicine, King Abdulaziz Medical City, Ministry of National Guard Health Affairs, Jeddah 21423, Saudi Arabia; 2 King Abdullah International Medical Research Centre, Jeddah 21423, Saudi Arabia; 3 College of Medicine, King Saud Bin Abdulaziz University for Health Sciences, Jeddah 21423, Saudi Arabia; 4 Department of Pathology and Laboratory Medicine, King Abdulaziz Medical City, Ministry of National Guard Health Affairs, Jeddah 21423, Saudi Arabia

**Keywords:** *Fusarium*, fusariosis, retrospective, invasive fungal infection, disseminated fungal infection, mycoses, Saudi Arabia

## Abstract

**Background/Objectives:** *Fusarium* species are recognized as difficult-to-treat opportunistic pathogens due to extensive antifungal resistance and high mortality rates. Variability in its incidence and outcomes exists across different countries and centers. Large studies on *Fusarium* species are lacking in Saudi Arabia, with most previous publications being case reports. We describe all cases of invasive fusariosis identified at a tertiary center during a 10-year period and review previous reports in the country. **Methods:** A retrospective search of hospital records and the microbiology database was conducted to identify cases of invasive fusariosis among patients admitted during 2016–2025 at King Abdulaziz Medical City, Jeddah, Saudi Arabia. **Results:** Three cases of invasive fusariosis occurring over a 10-year period were identified. All cases occurred in the last three years of the study period. The incidence during those three years was 0.4 cases per 10,000 admissions per year. Clinical manifestations were fungemia in two immunocompetent patients and ulcers progressing to osteomyelitis in an immunocompromised patient. None of the patients progressed to death within 30 days of diagnosis. **Conclusions:** Data on *Fusarium* species are scarce in Saudi Arabia. Additional studies are required to better understand differences in invasive fusariosis between countries.

## 1. Introduction

*Fusarium* spp. are a group of ubiquitous, filamentous, hyaline fungi that are emerging as difficult-to-treat opportunistic pathogens. The genus *Fusarium* has been recognized as a high research priority by the World Health Organization due to high antimicrobial resistance rates and the lack of large-scale studies [1]. Among immunocompetent patients, *Fusarium* species are usually responsible for superficial infections such as keratitis and onychomycosis [2]. Invasive fusariosis usually affects immunocompromised patients and can manifest as fungemia, cutaneous lesions, pneumonia, sinusitis, endophthalmitis, and disseminated infection [3]. Invasive fusariosis can also occur in immunocompetent patients in the context of severe burns or trauma, in patients receiving peritoneal dialysis, or in patients with long-term central venous catheters [4]. The incidence of invasive fusariosis varies between countries and hospitals and is highly dependent on the underlying conditions of the population being examined [4]. The highest incidence is typically observed among patients with hematological malignancy and those undergoing hematopoietic stem cell transplantation (HSCT) [3,4]. Culture-based diagnosis of *Fusarium* spp. is relatively reliable due to their propensity to cause fungemia and infections at surgically accessible sites [4]. Furthermore, *Fusarium* spp. can be easily identified by the production of characteristic sickle-shaped macroconidia that can be visualized with lactophenol staining [5]. *Fusarium* species are known for their poor in vitro susceptibility to most of the available antifungal agents, including amphotericin B and new triazoles [4,6]. However, antifungal susceptibility results have not been found to correlate with the survival rates of infection [6]. Clinical outcomes more often depend on recovery from neutropenia and the avoidance of steroid use [6]. There have been few studies on invasive fusariosis in Saudi Arabia. Only one retrospective cohort study was published from the country 25 years ago, which included seven patients with invasive infections, five of whom had disseminated infection [7]. The mortality rate in the aforementioned study was 71% after 30 days from the onset of infection [7]. In this study, we aimed to assess the occurrence of invasive fusariosis at a tertiary care center in the western region of Saudi Arabia and to review all reported cases from the country.

## 2. Materials and Methods

### 2.1. Study Setting

King Abdulaziz Medical City (KAMC) is a tertiary referral center and teaching hospital in the city of Jeddah, Saudi Arabia. It has a 751-bed capacity and admits patients from across the western region of Saudi Arabia. The center has a dedicated hematology and oncology division, a renal transplant unit, and a hematopoietic stem cell transplant unit. A specialized pediatric center within the medical city is also equipped with the same capabilities. Rooms serving patients with hematological malignancy and HSCT have positive-pressure ventilation systems and high-efficiency particulate air filters. There are many plant gardens that are open for patient access; however, bringing plants into patient rooms is restricted. Gardens in the hematology centers are gated behind closed glass windows, and patient access is not allowed.

### 2.2. Data Collection

We conducted a retrospective search of medical records and the microbiology database to identify cases of invasive fusariosis occurring among all patients treated at KAMC, Jeddah, over a 10-year period from 2016 to 2025. Records of all patients with a positive culture for *Fusarium* species were reviewed and categorized into proven, probable, or possible invasive fungal infection according to the revised European Organization for Research and Treatment of Cancer and Mycoses Study Group consensus definitions [8]. Possible cases were excluded, while all proven/probable cases were included and had information collected regarding age, sex, underlying illnesses, presence of central lines, receipt of immunosuppressive medications, presence of malignancy or neutropenia, history of HSCT, diagnostic procedures, treatments, and outcome 30 days from the diagnosis.

### 2.3. Diagnostic Procedures and Species Identification

Diagnostic procedures for all patients with invasive fusariosis included blood cultures, biopsy with histopathological examination of surgically accessible affected sites, ophthalmological examinations, and computed tomography (CT) scans of the chest, abdomen, pelvis, and sinuses. Blood samples were loaded into the BACTEC blood culture system (Becton Dickinson, Sparks, NC, USA), where they were incubated for 5 days. Tissue samples were inoculated on Sabouraud Dextrose Agar (Saudi Prepared Media Laboratory, Riyadh, Saudi Arabia) for 14 days and regularly examined for fungal growth. Repeat sampling was performed to rule out co-infection with other organisms. *Fusarium* species was identified based on the presence of sickle-shaped macroconidia under microscopy with lactophenol cotton blue staining. Further testing with matrix-assisted laser desorption ionization time-of-flight mass spectroscopy (Vitek MS, bioMérieux, Durham, NC, USA) was performed to identify the causative species. Therapeutic drug monitoring was performed for patients who received voriconazole, with a target concentration of 2–5 mcg/mL.

### 2.4. Definitions and Statistical Analysis

Neutropenia was defined as an absolute neutrophil count of less than 0.5 × 10^9^ cells/L. Disseminated infection was considered if there was involvement of two or more organs. The primary outcome was crude mortality at 30 days following the diagnosis of infection. Median and interquartile range (IQR) were used to present continuous variables, and frequencies (percentages) were used for categorical variables. Survival curves were constructed with the Kaplan–Meier method using JASP, version 0.19.3 (JASP Team 2024, University of Amsterdam, Amsterdam, The Netherlands).

## 3. Results

**Figure 1:**
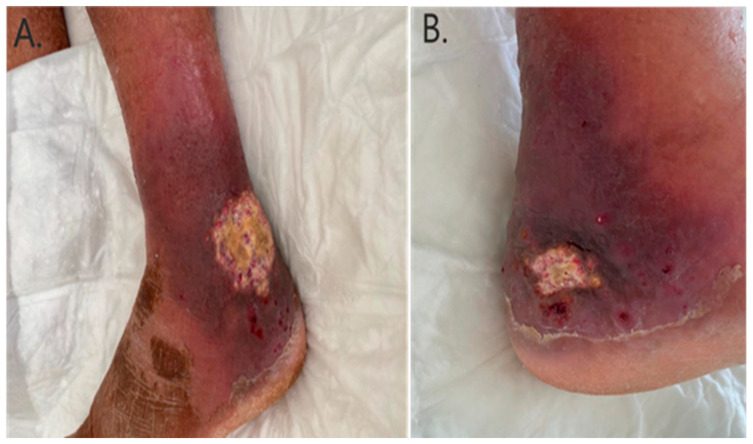
Cutaneous manifestations in Patient# 1. (**A**) A 6 × 6 cm, non-pyogenic, irregularly shaped ulcer over the lateral malleolus of the left foot, with a central yellowish crust and a surrounding zone of cellulitis with skin desquamation. (**B**) A 1.5 × 2 cm, non-pyogenic, irregular ulcer on the medial side of the left leg overlying the talar bone. Similar to the larger lesion, a yellowish central crust is present along with surrounding cellulitis and desquamation.

**Figure 2:**
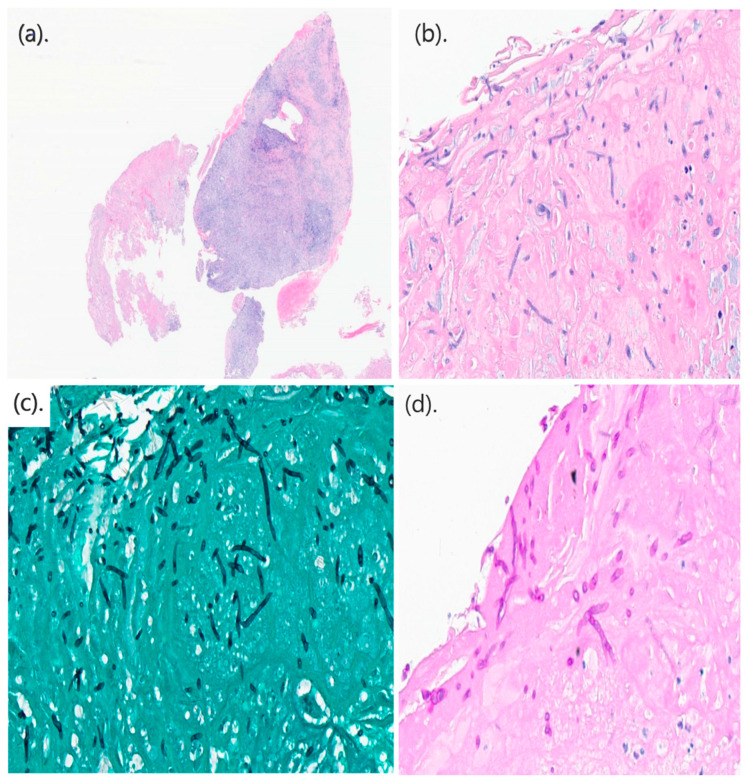
Histopathological findings in Patient# 1. (**a**) Low-power magnification showing severely ulcerated skin with prominent inflammation and granulation tissue (H&E, 20×). (**b**) Section of necrotic skin showing hyaline septate hyphae (H&E, 400×). (**c**) Grocott methenamine silver (GMS) stain highlighting numerous fungal hyphae in the subcutaneous tissue (400×). (**d**) Periodic acid–Schiff stain (PAS) showing septate hyaline hyphae of *F. chlamydosporum* (400×).

A total of three cases of invasive fusariosis were diagnosed at KAMC, Jeddah, between 2016 and 2025 (Table 1, Patient# 1–3). All cases occurred within the past three years of the study period at a rate of one case per year. During these three years, the hospital recorded 73,852 inpatient admissions, indicating an incidence of invasive fusariosis of 0.4 cases per 10,000 admissions per year. The median age of the three patients was 77 years, two of whom were elderly males, while one was a young adult female. Of the elderly patients, one was neutropenic secondary to acute myeloid leukemia (AML) and had chronic hepatitis B virus infection (Patient# 1). The other suffered from diabetes mellitus and interstitial lung disease (ILD) (Patient# 2). The remaining patient was in good health apart from living with sickle cell anemia (Patient# 3). Before being diagnosed with AML, Patient# 1, a farmer by occupation, developed small papules over his left leg without any obvious precipitating factors. He subsequently self-cauterized the lesions after two months, resulting in the development of ulceration and cellulitis (Figure 1). On presentation, *F. chlamydosporum* was isolated from multiple skin biopsies taken from the lesions, demonstrating positive histopathological findings (Figure 2). In addition, magnetic resonance imaging indicated underlying osteomyelitis affecting the talar bone and lateral malleolus of the left leg. Furthermore, chest CT showed bilateral lung nodules and ground-glass opacities; however, bronchoalveolar lavage was negative for bacteria, fungi, and mycobacteria. Cultures from the skin lesions grew *F. chlamydosporum*. Patient# 2 was admitted as a case of ILD exacerbation due to metapneumovirus infection three weeks before developing invasive fusariosis. On the current admission, he presented with subjective fever but was otherwise stable with regard to ILD. He was discharged on oral antibiotics after drawing peripheral blood cultures, which resulted in the growth of *F. oxysporum*. He was called back to undergo further investigations; however, no portal of entry or clinical manifestations other than fever were identified. Patient# 3 was hospitalized for over two months due to complications of sickle cell disease. Notably, she had central line-associated infections with multiple bacteria occurring at different intervals, despite line removal and appropriate antibiotics. A *Fusarium* species, identified only to the genus level by VITEK MS, was isolated from a peripherally inserted central catheter that had been placed five days prior. The patient had documented fever reaching 38.5 °C and concomitant infection with multiple bacteria, including *Escherichia hermannii*, *Ochrobactrum anthropi*, *Enterobacter cloacae*, *Comamonas aquatica*, and *Enterococcus casseliflavus*.

Patient# 1 received treatment with liposomal amphotericin B (LAMB) in combination with voriconazole (VOR) for 27 days, after which he requested transfer to a hospital in his home region. He was maintained on the same combination and was alive 42 days after the onset of infection. Patient# 2 initially received LAMB + VOR; however, VOR was discontinued after five days due to transaminitis. He continued LAMB monotherapy for 10 days, after which he was discharged on posaconazole tablets for 15 days. Regrettably, this patient passed away after completing the full course of treatment, 34 days after *Fusarium* fungemia. Death was considered to be due to his underlying condition, with unlikely contribution of fusariosis, as multiple blood cultures were negative; however, an autopsy was not performed. Patient# 3 received 28 days of VOR monotherapy and was alive more than a year after the onset of her infection.

**Figure 3:**
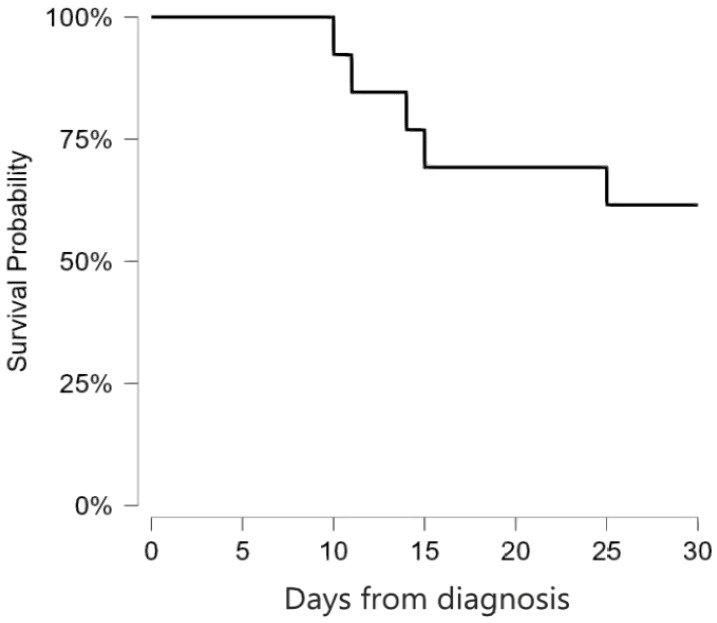
30-Day Kaplan–Meier survival curve for all patients with invasive fusariosis reported from Saudi Arabia (n = 13). All deaths occurred at least 10 days after diagnosis. The overall mortality rate was 38.4%.

Ten additional cases of invasive fusariosis in Saudi Arabia were identified through literature review, which are summarized in Table 1 [7,9,10,11,12]. Seven cases (70%) were reported before 1996 [7,9], while three cases (30%) occurred during the past 10 years, specifically in 2016 [12], 2017 [11], and 2020 [10]. Six patients were female, and four were male. The median age was 43 years (IQR: 29–51 years). Nine patients (90%) were immunocompromised, eight of whom had either leukemia or lymphoma (80%) [7,9,11], and one had a renal transplant (10%) [12]. The one immunocompetent patient experienced a central line-associated fungemia, where the line was inserted due to difficult peripheral access [10]. Species information was only available for the latter case and was *F. dimerum* [10]. Skin infection was the predominant clinical manifestation, affecting seven patients (70%). Disseminated infection was the second most common (60%), with involvement of skin, lungs, and eyes in one case [11], and unspecified involvement of two or more organs in five cases [7]. Central line-associated fungemia occurred in four cases (40%) [7,10]. The five patients with unspecified disseminated infection died within 30 days of diagnosis [7], while all other patients survived more than 30 days after the diagnosis. The mortality rate at 30 days was 50%. Figure 3 shows the Kaplan–Meier survival curve for all 13 cases reported in Saudi Arabia. Cases in the literature review are compared with the current study in Table 2.

## 4. Discussion

During this 10-year study, we observed only three cases of invasive fusariosis, all occurring within the last three years. In comparison, we previously reported 20 cases of trichosporonosis and 15 cases of mucormycosis during the same time period [13,14]. These species are similarly regarded as rare fungal pathogens and share risk factors for infection similar to *Fusarium* spp. [13,14]; however, invasive fusariosis demonstrated a significantly lower case burden in our experience.

The incidence of invasive fusariosis obtained in this study (0.4 cases/10,000 patients) appears comparable to that reported in other countries. For example, a study conducted in Santiago, Chile, investigating 268,188 hospital discharges between 2005 and 2015 also identified an incidence of invasive fusariosis of 0.4 cases/10,000 patients [15]. Another study in Madrid, Spain, investigating 858,150 admissions between 2004 and 2017, had a comparable incidence of approximately 0.1 cases/10,000 patients [16]. On the other hand, a study of the US Intermountain Healthcare network between 2006 and 2015 had a much lower incidence of 0.02 cases/10,000 patients [17]. Notably, incidence differs considerably when assessed in specific patient groups. In the aforementioned study in Madrid, the incidence among patients receiving antimould prophylaxis was much higher at 48.9 cases/10,000 patients compared to 0.1 cases/10,000 in the general population [16]. In contrast, a study of heart transplant recipients between 2010 and 2020 at the Cleveland Clinic, USA, identified one case of invasive fusariosis among 548 heart transplant recipients, equating to 18 cases/10,000 patients [18]. Predictably, the highest incidence was obtained in a study of patients with hematological malignancies across four Brazilian centers between 2015 and 2016, where three cases of invasive fusariosis occurred among 192 patients, equating to an incidence of 156 cases/10,000 patients [19].

Overall, cases in Saudi Arabia matched previous reports in several aspects. For instance, most patients (76%) who developed fusariosis were immunocompromised, mainly due to hematological malignancy (70%). However, only one patient in Saudi Arabia received an HSCT prior to *Fusarium* infection [9]. Skin infection was the most common clinical manifestation, affecting eight out of 13 patients (62%), all of whom had hematological malignancies. Five of the eight patients with cutaneous manifestations (62%) developed erythematous lesions with central necrosis, which are commonly associated with *Fusarium* infection [7]. The remaining skin manifestations were non-palpable purpura with flaccid central pustules (n = 1) and ulcerated skin lesions (n = 2) [11], Patient# 1. Cutaneous manifestations are an important part of *Fusarium* infection as they occur in more than 50% of infections compared with less than 10% in *Candida* or *Aspergillus* infections [20]. Disseminated infection was the second most common manifestation, affecting six out of thirteen patients (46%), followed by central line-associated fungemia in four out of thirteen cases (31%). All immunocompetent patients presented with isolated fungemia (n = 3) [10], Patients# 2–3, although contamination could not be definitively ruled out in the cases in this series due to single positive isolations. Mortality rates among cases from the literature review were 50%, matching the rates reported previously [3,4]; however, the addition of patients from this series, two of whom were immunocompetent, brought the overall mortality to 38%. Survival of *Fusarium* infection is strongly dependent on adequate neutrophil activity [6]; therefore, measures that boost neutrophil recovery, including granulocyte-stimulating factors and granulocyte transfusion, are occasionally employed in the treatment [9,11]. Two neutropenic patients from Saudi Arabia received granulocyte-stimulating factors, both of whom had a successful recovery [9,11]. However, the evidence supporting the use of these adjunct measures is not sufficient to be recommended to all neutropenic patients, especially with the risk of alloimmunization or severe transfusion-related reactions [21].

Genus-level identification of *Fusarium* species is usually uncomplicated due to the distinct shape of the macroconidia; however, species complex and species-level identification may be inaccurate in up to 50% of cases if identified using phenotypic methods [5]. Genetic sequencing is considered the definitive method for the identification of *Fusarium* isolates to the species level [5]; however, not all centers have access to this method. The use of the VITEK-MS system in this study enabled the identification of two out of three isolates to the species level, although the identification may not be concordant with the reference method for clinically rare species such as *F. chlamydosporum* [22]. On the other hand, only one other study in Saudi Arabia identified the reported *Fusarium* isolate to the species level; however, the identification method was not mentioned [10]. Susceptibility data are even more scarce, as none of the previous cases provided antifungal susceptibility results, including the current report, due to the lack of testing sources. This lack of information could greatly hamper future research efforts in specifying clinical breakpoints for susceptibility results.

Although we included all cases of invasive fusariosis at our center, the three cases identified represent a small sample with limited generalizability, which is also complicated by the single-center, retrospective design. Furthermore, reliance on culture-based identification misses possible culture-negative cases, and the lack of pan-fungal molecular testing directly on tissue samples means culture-negative involvement of certain organs could not be confirmed, and the possibility of co-infection could not be completely ruled out. The lack of susceptibility testing further limits the correlation of clinical success with microbiological characteristics of the isolates.

## 5. Conclusions

In conclusion, despite the low incidence identified, research on invasive fusariosis is lacking in Saudi Arabia. Regional surveillance and additional multicenter studies are needed to better identify the incidence across different areas in the country and to compare variability in clinical presentations with those reported in other countries.

## Figures and Tables

**Table 1:** Details of all invasive fusariosis cases reported from Saudi Arabia (n = 13).

Reference	(Current Study) Patient# 1	(Current Study) Patient# 2	(Current Study) Patient# 3	Musa & Ellis et al. [7,9]	Musa et al. [7] (n = 6)	Alshaya et al. [10]	AlShammasi et al. [11]	Alkhunaizi et al. [12]
Year	2023	2024	2022	1994	1985–1995	2020	2017	2016
City	Jeddah	Jeddah	Jeddah	Riyadh	Riyadh	Riyadh	Dammam	Dhahran
Region	Western	Western	Western	Central	Central	Central	Eastern	Eastern
Age	77	87	27	29	18–48	38	51	55
Sex	Male	Male	Female	Male	Female: 4 Male: 2	Female	Female	Male
Medical conditions	AML	ILD, DM, emphysema	SCA	NHL, Auto-HSCT	Hematological malignancy: 6	GI surgery	AML	Renal transplant
Immuno suppressive	Chemo	None	None	Chemo	Chemo: 6	None	Chemo	TAC, MMF
Organs affected	Skin, Bone, Lungs	Blood	Blood	Skin	Blood, Lungs: 1 N/A: 5	Blood	Skin, Lungs, Eye	Perinephric abscess
Species	*F. chlamydosporum*	*F. oxysporum*	N/A	N/A	N/A: 6	*F. dimerum*	N/A	N/A
Etiology	Self-cauterization	Unknown	Central line	Needle injury	Central line: 2	Central line	Unknown	Perinephric hematoma/ percutaneous drain
Treatment	LAMB, VOR	LAMB, VOR	VOR	LAMB, surgery, GMCSF	DAMB: 3 LAMB: 1 DAMB switched to LAMB: 2	LAMB, VOR	LAMB VOR, GCSF	VOR
Other infection (site)	HBV (liver)	Metapneumovirus (lungs)	Multiple bacteria (blood)	None	Gram negative sepsis: 1	*K. pneumoniae* (blood), COVID-19	None	Gram positive bacili
Death at day 30	No	No	No	No	Yes: 5	No	No	No

Data for all invasive fusariosis cases reported from Saudi Arabia (n = 13). Abbreviations: AML, acute myeloid leukemia; ILD, interstitial lung disease; DM, diabetes mellitus; SCA, sickle cell anemia; NHL, non-hodgkin lymphoma; Auto-HSCT, autologous hematopoietic stem cell transplant; GI, gastrointestinal; Chemo, chemotherapy; TAC, tacrolimus; MMF, mycophenolate mofetil; DAMB, deoxycholate amphotericin B; LAMB, liposomal amphotericin B; VOR, voriconazole; GCSF, granulocyte colony-stimulating factor; GMCSF, granulocyte monocyte colony-stimulating factor; HBV, hepatitis B virus; N/A, information not available.

**Table 2:** Comparison between cases in the current study and the literature review.

Variable	Current Study (n = 3)	Literature (n = 10)	Total (n = 13)
Study period:	2016–2025	1985–2020	1985–2025
Region of Saudi Arabia:			
Western region	3 (100%)	0 (0%)	3 (23%)
Central region	0 (0%)	8 (80%)	8 (62%)
Eastern region	0 (0%)	2 (20%)	2 (15%)
Age; median (IQR)	77 (N/A)	43 (29–51)	48 (28–66)
Male sex:	2 (66%)	4 (40%)	6 (46%)
Underlying condition:			
Leukemia/Lymphoma	1 (33%)	8 (80%)	9 (70%)
HSCT	0 (0%)	1 (10%); Autologous	1 (8%)
Solid organ transplant	0 (0%)	1 (10%); Renal	1 (8%)
Diabetes mellitus	1 (33%)	0 (0%)	1 (8%)
Immunosuppressives:			
Chemotherapy	1 (33%)	8 (80%)	9 (70%)
Steroids/TAC/MMF	0 (0%)	1 (10%)	1 (8%)
Isolated species:			
*F. chlamydosporum*	1 (33%)	0 (0%)	1 (8%)
*F. oxysporum*	1 (33%)	0 (0%)	1 (8%)
*F. dimerum*	0 (0%)	1 (10%)	1 (8%)
*Fusarium* spp.	1 (33%)	9 (90%)	10 (76%)
Portal of entry:			
Trauma	1 (33%)	1 (10%)	2 (15%)
Central line	1 (33%)	3 (30%)	4 (31%)
Transcutaneous drainage *	0 (0%)	1 (10%)	1 (8%)
Unknown	1 (33%)	5 (50%)	6 (46%)
Treatment:			
LAMB/VOR in combination	2 (66%)	2 (20%)	4 (31%)
VOR monotherapy	1 (33%)	1 (10%)	2 (15%)
LAMB monotherapy	0 (0%)	2 (20%)	2 (15%)
DAMB monotherapy	0 (0%)	3 (30%)	3 (23%)
DAMB switched to LAMB	0 (0%)	2 (20%)	2 (15%)
Adjunct therapy:			
Surgery	0 (0%)	1 (10%)	1 (8%)
GCSF or GMCSF	0 (0%)	2 (20%)	2 (15%)
Outcome at day 30:			
Alive	3 (100%)	5 (50%)	8 (62%)
Dead	0 (0%)	5 (50%)	5 (38%)

Data for invasive fusariosis cases from Saudi Arabia, comparing cases reported in the literature (n = 10) with cases from the current study (n = 3). Abbreviations: IQR, interquartile range; HSCT, hematopoietic stem cell transplant; TAC, tacrolimus; MMF, mycophenolate mofetil; DAMB, deoxycholate amphotericin B; LAMB, liposomal amphotericin B; VOR, voriconazole; GCSF, granulocyte colony-stimulating factor; GMCSF, granulocyte monocyte colony-stimulating factor. * Transcutaneous drainage of perinephric abscess. N/A, information not available.

## Data Availability

All data are contained in the article.

## Abbreviations

The following abbreviations are used in this manuscript:

AML	Acute myeloid leukemia
CT	Computed tomography
DAMB	Deoxycholate amphotericin B
DM	Diabetes mellitus
GCSF	Granulocyte colony stimulating factor
GMCSF	Granulocyte monocyte colony stimulating factor
GMS	Grocott methanamine silver stain
H&E	Hematoxylin and Eosin stain
HSCT	Hematopoietic stem cell transplant
HBV	Hepatitis B virus
ILD	Interstitial lung disease
IQR	Interquartile range
LAMB	Liposomal amphotericin B
PAS	Periodic acid schiff stain
MMF	Mycophenolate mofetil
NHL	Non-hodgkin lymphoma
TAC	Tacrolimus
VOR	Voriconazole
